# Adjunctive Value of Admission CBC-Derived Inflammation Indices for Catheter-Related Bloodstream Infection in Catheter-Dependent Hemodialysis Patients: A Retrospective Case–Control Study

**DOI:** 10.3390/diagnostics16121907

**Published:** 2026-06-19

**Authors:** Muhammed Ali Coşkuner, Gökhan Köker, Gülhan Özçelik Köker, Gizem Zorlu Görgülügil, Gökay Güven, Yasin Şahintürk, Bilgin Bahadır Başgöz, Ayça İnci, Derya Seyman

**Affiliations:** 1 Department of Internal Medicine, Antalya City Hospital, 07080 Antalya, Turkey; 2 Department of Internal Medicine, Antalya Training and Research Hospital, 07100 Antalya, Turkey; 3 Department of Medical Oncology, Faculty of Medicine, Akdeniz University, 07070 Antalya, Turkey; 4 Department of Nephrology, Antalya Training and Research Hospital, 07100 Antalya, Turkey; 5 Department of Infectious Diseases and Clinical Microbiology, Antalya Training and Research Hospital, 07100 Antalya, Turkey

**Keywords:** catheter-related infections, central venous catheters, renal dialysis, biomarkers

## Abstract

**Background/objectives:** Catheter-related bloodstream infection (CRBSI) is a frequent and morbid complication in catheter-dependent maintenance hemodialysis, and rapid risk stratification is needed while awaiting cultures. This study aimed to evaluate admission complete blood count-derived indices—neutrophil-to-lymphocyte ratio (NLR), lymphocyte-to-monocyte ratio (LMR), platelet-to-lymphocyte ratio (PLR), systemic immune-inflammation index (SII), and pan-immune-inflammation value (PIV)—for identifying CRBSI. **Methods:** This single-center retrospective study (1 January 2011–31 October 2024) included adult catheter-dependent hemodialysis patients classified as CRBSI or controls. CRBSI required compatible clinical findings and concordant growth of the same microorganism(s) in paired simultaneous catheter and peripheral blood cultures. Controls were hospitalized for non-infectious reasons without infection during the index admission. Indices were calculated from admission blood counts. Discrimination was assessed using ROC analysis, and adjusted associations were evaluated using multivariable logistic regression. **Results:** Among 286 patients (147 CRBSI, 139 controls), CRBSI cases had higher NLR, SII, and PIV and lower LMR; PLR did not differ. NLR showed the numerically highest discriminatory performance among the evaluated indices (AUC 0.737; cut-off 5.96; sensitivity 68.7%, specificity 68.3%; *p* < 0.001). SII (cut-off 1189.21; AUC 0.693) and PIV (cut-off 821.62; AUC 0.686) had moderate discrimination, and LMR was modest (cut-off 1.65; AUC 0.642); PLR was not discriminatory (AUC 0.559; *p* = 0.086). In models adjusted for age, sex, hypertension, and cardiovascular disease, NLR remained associated with CRBSI (OR 1.159; *p* < 0.001), together with hypertension (OR 2.441; *p* = 0.017) and cardiovascular disease (OR 2.626; *p* < 0.001). **Conclusions:** Admission hematologic inflammation indices, particularly NLR, showed moderate ability to discriminate CRBSI from non-infectious admissions in catheter-dependent hemodialysis patients and may provide rapid adjunctive information while awaiting microbiological confirmation.

## 1. Introduction

Hemodialysis (HD) patients remain disproportionately affected by bloodstream infections, which continue to drive substantial morbidity, hospitalization, and mortality in this population [1]. Vascular access is a key determinant of infectious risk, and central venous catheters—often required because of delayed or limited arteriovenous access options—are consistently associated with higher infection rates compared with fistulas or grafts and are a major contributor to dialysis-associated bloodstream infections [1,2,3]. In contemporary practice, 70–80% of patients initiate HD with a catheter, underscoring the ongoing clinical relevance of catheter-related complications and the need for robust preventive and diagnostic strategies [2,3,4].

Catheter-related bloodstream infection (CRBSI) is particularly consequential in HD because it can progress rapidly to sepsis, metastatic infection, and loss of vascular access, thereby increasing both patient-level burden and healthcare utilization. In dialysis facilities, bloodstream infections are recognized as a major safety threat, and system-level prevention frameworks (e.g., “core interventions”) have been emphasized because they can reduce dialysis-associated bloodstream infections—highlighting the magnitude and avoidability of this complication [2].

Accurate and timely diagnosis of CRBSI is essential to guide catheter management and antimicrobial therapy. Current guidance recommends microbiological confirmation using paired blood cultures obtained simultaneously from the catheter and a peripheral vein, interpreted alongside clinical assessment [5]. Diagnostic approaches such as differential time to positivity further support CRBSI diagnosis when paired cultures are obtained and processed under appropriate conditions [6]. However, culture-based confirmation is not immediately available at presentation, and clinical manifestations may be nonspecific in HD patients due to frequent comorbid inflammatory perturbations. As a result, there is a persistent need for readily available, objective markers that may support early identification and risk stratification at admission while definitive culture results are pending.

Complete blood count (CBC) parameters are routinely obtained at admission and may reflect host inflammatory responses in real time. Beyond single parameters, composite hematologic inflammation indices, such as the neutrophil-to-lymphocyte ratio (NLR), platelet-to-lymphocyte ratio (PLR), lymphocyte-to-monocyte ratio (LMR), systemic immune-inflammation index (SII), and pan-immune-inflammation value (PIV), have been proposed as integrative surrogates of systemic inflammation and immune status, incorporating leukocyte subsets and platelet activity. These indices have shown prognostic or predictive utility across diverse clinical settings and may offer a pragmatic bedside approach using universally available laboratory data [7,8]. In maintenance HD, NLR and related inflammatory metrics have recently been evaluated for CRBSI prediction, including models that combine CBC-derived indices with other inflammatory markers such as the CRP-to-albumin ratio [9].

Nevertheless, evidence remains limited regarding the comparative discriminatory performance of multiple CBC-derived indices measured at admission specifically for CRBSI in HD patients, and their relative utility after accounting for relevant clinical and laboratory covariates is not well established. This is particularly important because CRBSI risk in HD is multifactorial, influenced by patient characteristics, access-related factors, and broader clinical context [10]. Therefore, this study aimed to evaluate the adjunctive discriminatory value of hematologic inflammation indices measured at admission for identifying CRBSI in catheter-dependent HD patients. Unlike studies focusing on individual inflammatory markers or combined biomarker models, we comparatively assessed routinely available CBC-derived indices, including NLR, PLR, LMR, SII, and PIV, to determine which markers may provide the most feasible adjunctive information for early CRBSI suspicion at presentation. Rather than proposing these indices as novel biomarkers, this study was designed to clarify their relative and pragmatic adjunctive value in a narrowly defined catheter-dependent hemodialysis population with microbiologically confirmed CRBSI, a clinical context in which comparative admission-based data remain limited.

## 2. Materials and Methods

### 2.1. Study Design and Setting

This retrospective case–control study evaluating the adjunctive discriminatory value of admission hematologic inflammation indices was conducted at Antalya Training and Research Hospital, Turkey, and included adult patients receiving maintenance hemodialysis via a central venous catheter. Medical records of eligible patients managed between 1 January 2011, and 31 October 2024, were reviewed. Cases were patients with microbiologically confirmed CRBSI, whereas controls were catheter-dependent hemodialysis patients hospitalized for non-infectious indications without evidence of any infection during the index admission.

### 2.2. Ethical Approval

The study protocol was approved by the Antalya Training and Research Hospital Clinical Research Ethics Committee (Decision No: 18/17; date: 21 November 2024). Given the retrospective design and use of de-identified data, the requirement for informed consent was waived in accordance with institutional regulations and the Declaration of Helsinki.

### 2.3. Study Population and Eligibility Criteria

**Figure 1:**
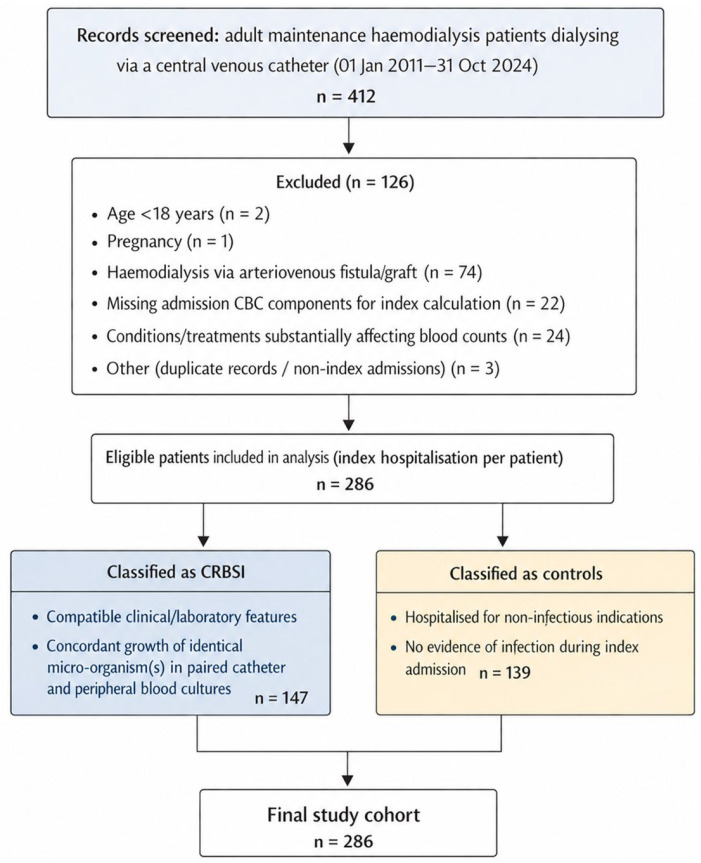
Flow diagram of patient screening, exclusions, and final cohort classification into CRBSI cases and controls among catheter-dependent maintenance hemodialysis admissions. CRBSI: catheter-related bloodstream infection; CBC, complete blood count.

A total of 412 records were screened (Figure 1). Patients were eligible if they met all of the following criteria: (i) age ≥ 18 years; (ii) end-stage kidney disease on routine maintenance hemodialysis via a central venous catheter; and (iii) availability of admission complete blood count (CBC) parameters required to calculate the hematologic inflammation indices.

Patients were excluded for the following reasons (Figure 1): age < 18 years (*n* = 2), pregnancy (n = 1), hemodialysis via an arteriovenous fistula or graft (n = 74), missing admission CBC components required for index calculation (*n* = 22), and conditions or treatments expected to substantially affect admission blood counts (*n* = 24; e.g., autoimmune disease, hematologic disorders, advanced liver failure, or medications known to influence blood cell counts). Additional exclusions included duplicate records or non-index admissions (*n* = 3). After exclusions (*n* = 126), 286 eligible patients were included in the final analysis.

CRBSI group (cases): Patients were classified as CRBSI cases if they had compatible clinical and/or laboratory features suggestive of catheter-related infection together with microbiological confirmation. Microbiological confirmation was defined as concordant growth of the same microorganism(s) in paired blood cultures obtained simultaneously from the catheter and a peripheral vein. Paired catheter and peripheral blood cultures were routinely obtained before initiation of empiric antimicrobial therapy when CRBSI was suspected, in accordance with institutional clinical practice. In total, 147 patients met the criteria for CRBSI (Figure 1).

Control group: Controls were selected from the same institutional catheter-dependent maintenance hemodialysis population and the same overall study period. They consisted of catheter-dependent hemodialysis patients hospitalized for non-infectious indications, without clinical, laboratory, or microbiological evidence of infection at admission or during the early index hospitalization. Where documented, controls also had no initiation of systemic antibiotics for suspected infection. Controls were not individually matched to CRBSI cases according to calendar year, dialysis vintage, catheter type, catheter insertion site, or catheter dwell time, because these variables were not consistently and reliably available in the retrospective records. Therefore, the control group should be interpreted as a non-infectious hospitalized catheter-dependent hemodialysis comparator group rather than as a matched clinical-suspicion cohort. A total of 139 patients were included as controls (Figure 1).

### 2.4. Definitions

CRBSI was defined by the presence of compatible clinical and/or laboratory features together with microbiological confirmation—specifically, paired blood cultures obtained simultaneously from the catheter and peripheral blood yielding the same microorganism(s)—consistent with established diagnostic principles for intravascular catheter-related infection.

### 2.5. Data Collection

Demographic characteristics and comorbidities, including diabetes mellitus, hypertension, and cardiovascular disease, were abstracted from medical records. Admission laboratory data collected at presentation/hospitalization included white blood cell count (WBC), hemoglobin, platelet count, neutrophil, lymphocyte, and monocyte counts, mean platelet volume (MPV), and C-reactive protein (CRP). All measurements were obtained from the first blood samples drawn at hospital admission or at the time of initial clinical evaluation, before culture results became available. In patients classified as CRBSI, these laboratory measurements corresponded to the admission evaluation performed at the time of clinical suspicion for catheter-related infection. Therefore, the hematologic indices were assessed for their ability to discriminate patients with microbiologically confirmed CRBSI from non-infectious catheter-dependent hemodialysis controls at presentation. They were not evaluated as longitudinal predictors of future CRBSI development in initially infection-free patients.

### 2.6. Hematologic Inflammation Indices

Indices were calculated using admission CBC parameters as follows:

NLR = Neutrophils (×10^9^/L)/Lymphocytes (×10^9^/L)

LMR = Lymphocytes (×10^9^/L)/Monocytes (×10^9^/L)

PLR = Platelets (×10^9^/L)/Lymphocytes (×10^9^/L)

SII = [Platelets (×10^9^/L) × Neutrophils (×10^9^/L)]/Lymphocytes (×10^9^/L)

PIV = [Platelets (×10^9^/L) × Neutrophils (×10^9^/L) × Monocytes (×10^9^/L)]/Lymphocytes (×10^9^/L)

### 2.7. Study Endpoint

The study endpoint was the ability of admission hematologic inflammation indices (NLR, LMR, PLR, SII, and PIV) to discriminate microbiologically confirmed CRBSI cases from non-infectious catheter-dependent hemodialysis control admissions.

### 2.8. Statistical Analysis

Statistical analysis was performed using SPSS (IBM Corporation, Armonk, NY, USA), version 27.0. Data distribution was assessed using the Shapiro–Wilk test. For parametric variables, data were expressed as mean (standard deviation), and Student’s *t*-test was used for comparisons. Non-parametric variables were presented as median (interquartile range [IQR]), and the Mann–Whitney U test was used, as appropriate. Categorical variables were expressed as number (%) and compared using the chi-square test. To evaluate the discriminatory performance of admission inflammatory markers for CRBSI, receiver operating characteristic (ROC) curve analysis was performed, and area under the curve (AUC) values were calculated. The primary CBC-derived composite hematologic indices included NLR, LMR, PLR, SII, and PIV. In additional exploratory ROC analyses, conventional inflammatory markers available at admission, including WBC count, neutrophil count, and CRP, were also evaluated to contextualize the discriminatory performance of the CBC-derived composite indices. Optimal cut-off values were determined using the Youden index, and corresponding sensitivity and specificity values were reported. These cut-off values were considered exploratory and cohort-specific because no internal or external validation cohort was available. Point-biserial correlation was calculated to assess the association between admission inflammatory markers and CRBSI. Pairwise comparisons of AUC values between selected CBC-derived composite indices (NLR, SII, and PIV) and conventional inflammatory markers (WBC count, neutrophil count, and CRP) were performed using the MedCalc online calculator for comparison of independent ROC curves, version 23.6.1 (MedCalc Software Ltd., Ostend, Belgium). Differences between AUCs were reported as ΔAUC with standard error (SE) and corresponding *p* values.

To evaluate variables associated with CRBSI status, univariate binary logistic regression analyses were performed using the Enter method. In addition to age and sex, variables that were statistically significant in the univariate analyses were further evaluated in multivariable binary logistic regression models. Before multivariable logistic regression, multicollinearity among independent variables was assessed, and variables with multicollinearity above the acceptable level (r > 0.70 or variance inflation factor [VIF] > 3) were excluded. For improved interpretability in logistic regression models, SII and PIV were expressed per 100-unit increase because these indices are measured on a scale of hundreds to thousands. Nagelkerke R-square values were reported for each regression model. Statistical significance was set at *p* < 0.05, and analyses were conducted at the 95% confidence level. No imputation was performed. Patients with missing admission CBC components required for calculation of the hematologic indices were excluded from the analysis, as shown in Figure 1.

## 3. Results

A total of 286 maintenance hemodialysis patients dialyzing via a central venous catheter were included, comprising 147 patients with CRBSI and 139 controls (Table 1). Age and sex distributions were comparable between groups, whereas hypertension and cardiovascular disease were more frequent in the CRBSI group (Table 1).

At admission, the CRBSI group demonstrated a markedly higher inflammatory profile, including elevated WBC and CRP compared with controls (Table 1). Hemogram-derived indices also differed substantially: NLR, SII, and PIV were significantly higher in the CRBSI group, whereas LMR was significantly lower; PLR did not show a significant between-group difference (Table 1).

Point-biserial correlation analysis showed that CRP had the strongest association with CRBSI among the evaluated admission inflammatory markers (r = 0.714, *p* < 0.001), followed by neutrophil count (r = 0.447, *p* < 0.001), WBC count (r = 0.411, *p* < 0.001), and NLR (r = 0.411, *p* < 0.001). Among the CBC-derived composite indices, NLR had the highest correlation coefficient, although the magnitude of association was moderate. Additional positive correlations were observed for SII (r = 0.333, *p* < 0.001) and PIV (r = 0.323, *p* < 0.001), whereas LMR showed a negative correlation with CRBSI (r = −0.246, *p* < 0.001), indicating that lower LMR values were associated with CRBSI (Table 2).

**Figure 2:**
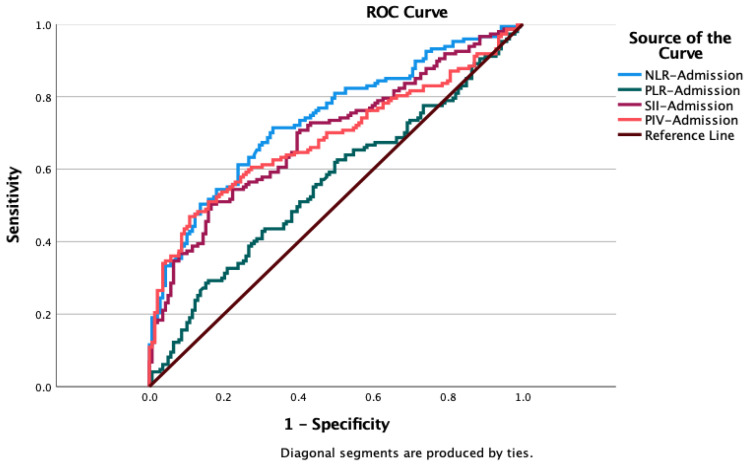
ROC curves of NLR, PLR, SII, and PIV at admission for discriminating CRBSI from non-infectious control admissions in catheter-dependent hemodialysis patients. Abbreviations: ROC, receiver operating characteristic; NLR, neutrophil-to-lymphocyte ratio; PLR, platelet-to-lymphocyte ratio; SII, systemic immune-inflammation index; PIV, pan-immune-inflammation value.

**Figure 3:**
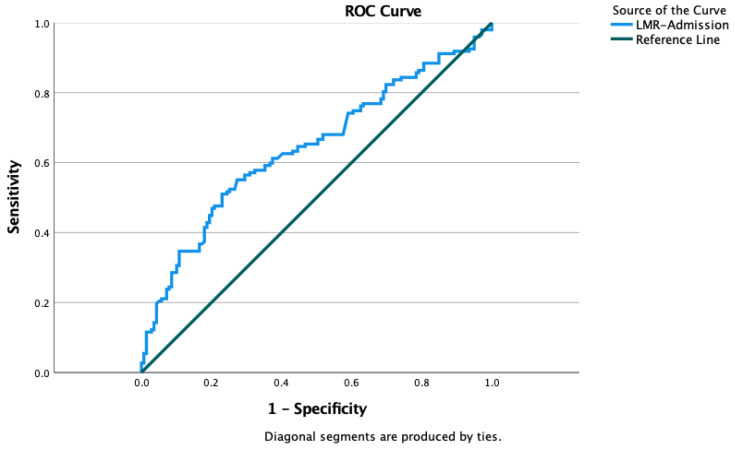
ROC curve of LMR at admission for discriminating CRBSI from non-infectious control admissions in catheter-dependent hemodialysis patients. Abbreviations: ROC, receiver operating characteristic; LMR, lymphocyte-to-monocyte ratio.

ROC analyses demonstrated that CRP had the highest discriminatory performance for CRBSI among all evaluated admission inflammatory markers (AUC 0.913, 95% CI 0.879–0.947; *p* < 0.001), with an optimal cut-off of 72.5 mg/L, sensitivity of 83.7%, and specificity of 83.5%. Neutrophil count also demonstrated moderate-to-good discrimination (AUC 0.756, 95% CI 0.700–0.813; *p* < 0.001), with a cut-off of 6.59 × 10^9^/L, sensitivity of 72.1%, and specificity of 69.1%. WBC count showed discriminatory performance similar to NLR (AUC 0.737, 95% CI 0.679–0.795; *p* < 0.001), with a cut-off of 8.85 × 10^9^/L, sensitivity of 69.4%, and specificity of 66.2%. Among the CBC-derived composite indices, NLR had the numerically highest AUC (AUC 0.737, 95% CI 0.680–0.795; *p* < 0.001), with an optimal cut-off of 5.96, sensitivity of 68.7%, and specificity of 68.3%. SII and PIV also showed moderate discriminatory ability, and LMR showed modest inverse discrimination, whereas PLR did not demonstrate statistically significant discrimination (Table 2; Figure 2 and Figure 3). Pairwise ROC AUC comparisons were performed to compare CBC-derived composite indices with conventional inflammatory markers. NLR, SII, and PIV did not show statistically significant AUC differences compared with WBC count or neutrophil count (all *p* > 0.05). However, all three indices had significantly lower AUCs than CRP (NLR vs. CRP: ΔAUC 0.183, SE 0.035, *p* < 0.001; SII vs. CRP: ΔAUC 0.227, SE 0.036, *p* < 0.001; PIV vs. CRP: ΔAUC 0.232, SE 0.037, *p* < 0.001) (Table 3).

In univariate logistic regression, hypertension (OR 2.336, *p* = 0.003) and cardiovascular disease (OR 2.977, *p* < 0.001) were significantly associated with CRBSI. Among the CBC-derived hematologic indices, NLR was significantly associated with CRBSI (OR 1.167, 95% CI 1.104–1.233; *p* < 0.001), and SII and PIV also showed significant associations when expressed per 100-unit increase (Table 4).

To address collinearity among CBC-derived indices, NLR, SII, and PIV were evaluated in separate multivariable models adjusted for age, sex, hypertension, and cardiovascular disease. In these exploratory models, each index remained statistically associated with CRBSI. In the NLR model, NLR remained independently associated with CRBSI (OR 1.159, 95% CI 1.099–1.223; *p* < 0.001), together with hypertension (OR 2.441, *p* = 0.017) and cardiovascular disease (OR 2.626, *p* < 0.001) (Table 5). In the SII model, SII remained significantly associated with CRBSI when expressed per 100-unit increase (OR 1.056, 95% CI 1.033–1.079; *p* < 0.001), together with hypertension (OR 2.014, *p* = 0.046) and cardiovascular disease (OR 2.702, *p* < 0.001) (Table 6). Similarly, in the PIV model, PIV remained significantly associated with CRBSI when expressed per 100-unit increase (OR 1.063, 95% CI 1.036–1.090; *p* < 0.001), together with hypertension (OR 2.205, *p* = 0.030) and cardiovascular disease (OR 2.730, *p* < 0.001) (Table 7). Overall, model fit was moderate (Nagelkerke R^2^ range: 0.259–0.322; Table 5, Table 6 and Table 7).

## 4. Discussion

In this retrospective case–control study of maintenance hemodialysis patients dialyzing via central venous catheters, admission inflammatory markers demonstrated varying discriminatory performance for CRBSI. The main findings can be summarized as follows: (i) CRP showed the highest overall discriminatory performance among the evaluated admission inflammatory markers; (ii) among CBC-derived composite hematologic indices, NLR showed the numerically most favorable discriminatory profile, although its performance remained moderate and below the level generally considered to indicate strong discrimination (AUC 0.737; sensitivity 68.7%, specificity 68.3%); (iii) SII and PIV demonstrated moderate discriminatory performance, LMR was inversely associated with CRBSI, and PLR was not discriminatory; and (iv) hypertension and cardiovascular disease were consistently associated with CRBSI in adjusted exploratory models (Table 1, Table 2, Table 3, Table 4, Table 5, Table 6 and Table 7; Figure 2 and Figure 3). Pairwise AUC comparisons showed that NLR, SII, and PIV did not significantly differ from WBC count or neutrophil count, whereas all three had significantly lower AUCs than CRP (Table 3). These findings suggest that CBC-derived hematologic indices may provide rapid adjunctive information at admission, but they should not be interpreted as replacements for conventional inflammatory markers, clinical assessment, or microbiological confirmation. However, the observed AUC values should not be interpreted as sufficient stand-alone diagnostic performance. Rather, these indices may complement early clinical assessment and microbiological sampling, but they cannot confirm or exclude CRBSI in isolation.

The lack of significant discriminatory performance for PLR may be explained by the complex behavior of platelet-related parameters in end-stage kidney disease and acute infection. Although platelet and lymphocyte counts showed between-group trends, their combined ratio did not amplify the inflammatory signal sufficiently to provide meaningful discrimination. In patients receiving hemodialysis, platelet counts and platelet-related indices may be influenced by uremia-associated platelet dysfunction, dialysis-related factors, medications, inflammation-related platelet consumption, and comorbidity burden. Therefore, PLR may be less specific for CRBSI than indices that incorporate neutrophil predominance and relative lymphopenia, such as NLR, or broader composite inflammatory formulas such as SII and PIV. SII and PIV were included because they represent expanded CBC-derived composite indices that integrate neutrophil, lymphocyte, platelet, and, for PIV, monocyte components of the inflammatory response. Although these indices are less established in nephrology than NLR, they were evaluated as exploratory markers to compare whether broader host-response indices could provide additional discriminatory information in catheter-dependent hemodialysis patients. The present findings should therefore be interpreted as exploratory and hypothesis-generating rather than as evidence that SII and PIV are established nephrology-specific diagnostic biomarkers. MPV was reported only as a routine platelet-related CBC parameter for descriptive characterization and was not treated as a predefined composite hematologic inflammation index in the primary analyses.

The favorable discriminatory profile of NLR among CBC-derived composite indices is biologically plausible and aligns with emerging evidence in hemodialysis populations. Infections in dialysis patients often present with attenuated or nonspecific symptoms; however, acute bacterial inflammation typically shifts leukocyte subsets toward neutrophilia and relative lymphopenia, producing a higher NLR. Prior work in hemodialysis has shown that combining CRP with NLR improves the identification of infectious inflammation at admission, highlighting the additive value of pairing routine inflammatory biomarkers with leukocyte-derived ratios [11]. In the CRBSI-specific setting, Zhao et al. reported that NLR, particularly when combined with the CRP-to-albumin ratio, was useful for predicting CRBSI among maintenance hemodialysis patients, reinforcing the idea that leukocyte-ratio-based metrics capture early inflammatory signals relevant to catheter infections [9]. Our findings extend this literature by providing a comparative assessment of multiple CBC-derived indices measured at admission, while also contextualizing their performance against conventional inflammatory markers. The additional ROC analyses showed that CRP had clearly superior discriminatory performance compared with NLR, SII, and PIV in this case–control cohort, which is expected given the comparison between microbiologically confirmed CRBSI cases and non-infectious controls. In contrast, NLR showed discriminatory performance comparable to WBC count and not significantly different from neutrophil count. This interpretation is also consistent with recent hemodialysis CRBSI studies showing that infectious episodes are frequently accompanied by systemic inflammatory activation and that routinely available clinical and laboratory variables may help characterize early risk before culture confirmation [12,13,14]. Therefore, the present findings do not support the use of CBC-derived indices as replacements for CRP or other conventional inflammatory markers. Instead, they define a more limited but clinically relevant role for these indices: rapidly available, low-cost adjunctive markers derived from routine CBC parameters that may contribute additional host-response information when interpreted together with CRP, WBC count, neutrophil count, catheter assessment, clinical presentation, and microbiological sampling while culture confirmation is pending.

Beyond NLR, SII and PIV integrate platelet counts into the inflammatory signal. Platelets can participate in antimicrobial defense and thromboinflammation, and platelet activation is increasingly recognized as part of the systemic response to infection. Indices incorporating platelets therefore may reflect a broader inflammatory phenotype than leukocyte ratios alone. While our study focused on diagnostic discrimination rather than long-term outcomes, the recent hemodialysis literature supports the clinical relevance of these composite indices: Elevated SII has been associated with infection-related and all-cause mortality among hemodialysis patients, suggesting that higher systemic inflammatory burden measured by SII is clinically consequential in this population [15]. Similarly, PIV has been investigated in maintenance hemodialysis cohorts and linked to adverse outcomes, supporting its role as a systemic inflammatory marker in dialysis settings [16]. Recent outcome-based studies in dialysis and catheter-dependent populations further support the concept that composite inflammation indices may capture a broader host-response phenotype rather than serving as pathogen-specific markers [17,18]. Therefore, the moderate performance of SII and PIV in the present study should be interpreted as evidence of systemic inflammatory burden associated with CRBSI rather than as direct microbiological diagnostic markers. Collectively, these data provide external biologic and clinical context for why SII and PIV showed moderate discriminatory performance for CRBSI at admission in our cohort.

The inverse association observed for LMR may reflect the combined effects of lymphopenia during acute infection and relative expansion and activation of the monocyte compartment. Because monocytes are key mediators of innate immunity and cytokine signaling, shifts in the monocyte-to-lymphocyte balance may capture infection-related immune dysregulation. In contrast, PLR did not demonstrate adjunctive discriminatory value in our cohort. This may relate to the complex platelet biology in end-stage kidney disease—characterized by uremia-associated platelet dysfunction and variable platelet activation, and further influenced by comorbidities, medications, and inflammation-related platelet consumption—which can reduce the specificity of platelet-based ratios for detecting infection at a single time point [19,20]. Although MPV differed significantly between groups, it was reported only as a routine platelet-related CBC parameter for descriptive characterization. MPV was not considered one of the predefined composite hematologic inflammation indices and was not included in the primary ROC or multivariable index-based analyses. Therefore, this finding should be considered descriptive and hypothesis-generating.

From a clinical risk perspective, the consistent association of hypertension and cardiovascular disease with CRBSI in adjusted models is concordant with the multifactorial risk architecture described in the broader hemodialysis CRBSI literature. These associations should be interpreted cautiously and may reflect overall comorbidity burden rather than direct causal effects. A recent meta-analysis identified multiple contributors to catheter-associated bloodstream infection in hemodialysis—spanning patient factors, including comorbidities, access-related variables, and inflammatory biomarkers—underscoring that CRBSI risk is shaped by both host vulnerability and catheter-related exposures [10]. Although our analysis was not designed to adjudicate causal pathways for comorbidity associations, these findings emphasize that CBC-derived indices should be interpreted within a broader clinical context rather than as stand-alone diagnostics. This point is clinically relevant because recent hemodialysis cohorts have shown that CRBSI risk and outcomes are influenced not only by inflammatory status but also by catheter-related factors, local microbiological patterns, patient frailty, and comorbidity burden [12,13,14,21,22,23]. The inverse association of age observed only in the NLR-adjusted model should also be interpreted cautiously, as age was not associated with CRBSI in univariate analysis and this association was not consistent across the adjusted models. This model-specific finding may reflect adjustment or suppression effects after inclusion of NLR and comorbidity variables rather than a robust independent clinical association. Accordingly, CBC-derived indices may be most useful when incorporated into a broader bedside assessment that includes vascular access characteristics, clinical presentation, comorbidity profile, conventional inflammatory markers, and microbiological sampling strategy.

Clinically, the results suggest a pragmatic adjunctive approach: admission NLR, supported by indices such as SII or PIV when available, may help prioritize suspicion for CRBSI in catheter-dependent hemodialysis patients presenting with nonspecific symptoms, potentially prompting timely paired culture acquisition, closer monitoring, and earlier alignment with established catheter-infection prevention and management principles [24]. However, these indices should be interpreted alongside clinical assessment and should not be used as stand-alone diagnostic tests [9]. Importantly, these indices are not substitutes for microbiological confirmation; rather, they may function as rapid, low-cost adjuncts to guide early decision-making while awaiting definitive culture results.

Several limitations should be acknowledged. First, the retrospective single-center design introduces a risk of selection bias, residual confounding, and limited generalizability. Second, the control group consisted of non-infectious hospitalized catheter-dependent hemodialysis patients rather than patients with suspected CRBSI or alternative infectious/inflammatory conditions. In addition, controls were not individually matched to CRBSI cases according to calendar period, dialysis vintage, catheter type, catheter insertion site, or catheter dwell time, because these variables were not consistently available in the retrospective records. Therefore, the present case–control comparison reflects discrimination between microbiologically confirmed CRBSI and clearly non-infectious admissions, rather than diagnostic performance in an unselected real-world population with clinical suspicion of CRBSI. This design may have overestimated the apparent discriminatory performance of the evaluated indices, and the reported AUC values should be interpreted cautiously. Third, detailed catheter-related and clinical variables, including catheter type, insertion site, dwell time, previous CRBSI, catheter care or lock protocols, primary kidney disease, serum albumin, malignancy, immunosuppressive therapy, and steroid exposure, were not consistently available and could not be incorporated into the regression models. Residual confounding from unmeasured catheter-related and host-related factors therefore cannot be excluded. Fourth, the long study period may have introduced temporal heterogeneity related to changes in catheter care practices, infection prevention protocols, microbiological workflows, and antimicrobial management over time. Although a sensitivity analysis by calendar period could theoretically address this issue, reliable protocol-level information regarding the timing and implementation of changes in catheter care, culture processing, and antimicrobial practice was not consistently available. Therefore, dividing the cohort into arbitrary time periods would not necessarily reflect clinically meaningful practice changes and could introduce additional instability. The potential influence of temporal changes should therefore be considered an important limitation. Fifth, although a stringent microbiological definition based on simultaneous paired catheter and peripheral blood cultures was used, additional diagnostic refinements such as differential time to positivity were not available for all cases. Paired cultures were routinely obtained before empiric antimicrobial therapy according to institutional practice; however, the exact timing of culture acquisition and antibiotic initiation could not be independently verified for every admission because of the retrospective design. Sixth, detailed microbiological data, including pathogen distribution, Gram-positive and Gram-negative organism classification, coagulase-negative staphylococci frequency, antimicrobial resistance patterns, infection severity, and clinical outcomes, were not consistently available in sufficient detail for inclusion in the primary analysis. This may limit the microbiological interpretability and overall clinical relevance of the findings. Finally, the ROC-derived cut-off values were not internally or externally validated and should be considered exploratory and cohort-specific. These thresholds should not be directly applied in clinical practice without prospective validation in clinically suspected CRBSI populations that include infectious and inflammatory comparator groups.

## 5. Conclusions

In conclusion, admission CBC-derived hematologic inflammation indices showed moderate ability to discriminate microbiologically confirmed CRBSI cases from non-infectious catheter-dependent hemodialysis control admissions. Among these indices, NLR demonstrated the most favorable discriminatory profile; however, CRP showed substantially stronger overall performance among the evaluated admission inflammatory markers. Therefore, the present findings do not demonstrate incremental diagnostic value beyond CRP, and CBC-derived indices should not be interpreted as replacements for CRP, clinical assessment, or microbiological confirmation. Rather, they may provide rapid, low-cost adjunctive host-response information derived from routine CBC parameters during initial clinical evaluation. Their performance is insufficient for stand-alone diagnosis or exclusion of CRBSI, and paired blood cultures with microbiological confirmation remain essential. Because this study used a case–control design with non-infectious controls, these exploratory findings require prospective validation in clinically suspected CRBSI populations that include infectious and inflammatory comparator groups.

## Figures and Tables

**Table 1:** Baseline characteristics and admission laboratory findings of hemodialysis patients with and without catheter-related bloodstream infection.

	Total (*n* = 286)	CRBSI Group (*n* = 147)	Control Group (*n* = 139)	*p*
Age, years, median (IQR)	63 (22)	63 (23)	64 (21)	0.892
Female sex, n (%)	145 (50.7)	79 (53.7)	66 (47.5)	0.290
Comorbid conditions				
Diabetes mellitus, n (%)	135 (47.2)	69 (46.9)	66 (47.5)	0.927
Hypertension, n (%)	216 (75.5)	122 (83)	94 (67.6)	0.003
Cardiovascular disease, n (%)	142 (49.7)	92 (62.6)	50 (36)	<0.001
Laboratory Findings on Admission				
WBC (×10^9^/L), median (IQR)	8.95 (6.43)	11.50 (8.10)	7.70 (3.90)	<0.001
Hemoglobin (g/dL), median (IQR)	9.40 (2.63)	9.80 (2.50)	8.90 (2.50)	0.004
Platelets (×10^9^/L), median (IQR)	184.5 (114.0)	170.0 (120.0)	196.0 (112.0)	0.072
Neutrophils (×10^9^/L), median (IQR)	6.76 (5.85)	8.70 (7.30)	5.61 (3.20)	<0.001
Lymphocytes (×10^9^/L), median (IQR)	1.10 (0.88)	1.10 (1.00)	1.20 (0.80)	0.018
Monocytes (×10^9^/L), median (IQR)	0.70 (0.50)	0.80 (0.70)	0.64 (0.36)	0.005
NLR, median (IQR)	5.98 (7.59)	9.00 (10.79)	4.25 (4.18)	<0.001
LMR, median (IQR)	1.66 (1.32)	1.33 (1.29)	1.82 (1.22)	<0.001
PLR, median (IQR)	173.65 (160.0)	185.45 (189.67)	159.65 (124.77)	0.086
SII, median (IQR)	1193.16 (1490.56)	1819.75 (2673.00)	867.93 (951.62)	<0.001
PIV, median (IQR)	833.92 (1429.87)	1312.58 (2544.10)	623.18 (692.59)	<0.001
MPV (fL), mean (SD)	9.96 (1.40)	9.29 (1.42)	10.70 (0.91)	<0.001
CRP (mg/L), median (IQR)	77.5 (145.0)	144.0 (137.0)	16.0 (44.0)	<0.001

CRBSI, catheter-related bloodstream infection; n, number; IQR, interquartile range; WBC, white blood cell count; NLR, neutrophil-to-lymphocyte ratio; LMR, lymphocyte-to-monocyte ratio; PLR, platelet-to-lymphocyte ratio; SII, systemic immune-inflammation index; PIV, pan-immune-inflammation value; MPV, mean platelet volume; CRP, C-reactive protein; SD, standard deviation.

**Table 2:** Correlation and discriminatory performance of admission inflammatory markers for catheter-related bloodstream infection in hemodialysis patients.

	Point-Biserial Correlation		ROC				
	r	*p*	Cut-Off	Sensitivity (%)	Specificity (%)	AUC, 95% CI	*p*
NLR	0.411	<0.001	5.96	68.7	68.3	0.737, 0.680–0.795	<0.001
LMR	−0.246	<0.001	1.65	61.2	62.6	0.642, 0.578–0.706	<0.001
PLR	0.102	0.086	172.24	55.8	55.4	0.559, 0.492–0.625	0.086
SII	0.333	<0.001	1189.21	63.3	63.3	0.693, 0.632–0.753	<0.001
PIV	0.323	<0.001	821.62	63.9	63.3	0.686, 0.624–0.749	<0.001
WBC count	0.411	<0.001	8.85	69.4	66.2	0.737, 0.679–0.795	<0.001
Neutrophil count	0.447	<0.001	6.59	72.1	69.1	0.756, 0.700–0.813	<0.001
CRP	0.714	<0.001	72.5	83.7	83.5	0.913, 0.879–0.947	<0.001

**Table 3:** Pairwise comparisons of ROC AUCs between CBC-derived composite indices and conventional inflammatory markers.

	WBC Count		Neutrophil Count		CRP	
	ΔAUC (SE)	*p*	ΔAUC (SE)	*p*	ΔAUC (SE)	*p*
NLR	0.007 (0.042)	0.869	0.026 (0.042)	0.533	0.183 (0.035)	<0.001
SII	0.051 (0.043)	0.237	0.070 (0.042)	0.099	0.227 (0.036)	<0.001
PIV	0.056 (0.044)	0.202	0.075 (0.043)	0.082	0.232 (0.037)	<0.001

ΔAUC was calculated as the AUC of the conventional inflammatory marker minus the AUC of the CBC-derived composite index. Positive values indicate a higher AUC for the conventional marker. AUC, area under the curve; CBC, complete blood count; CRP, C-reactive protein; NLR, neutrophil-to-lymphocyte ratio; PIV, pan-immune-inflammation value; ROC, receiver operating characteristic; SE, standard error; SII, systemic immune-inflammation index; WBC, white blood cell count.

**Table 4:** Univariate logistic regression analysis of clinical variables and hematologic inflammation indices for catheter-related bloodstream infection in hemodialysis patients.

	Univariate Analysis				
	β	OR	95% CI		*p*
			Lower	Upper	
Age	0.002	1.002	0.987	1.016	0.842
Female sex	0.251	1.285	0.807	2.045	0.290
Diabetes mellitus	−0.022	0.978	0.615	1.557	0.927
Hypertension	0.849	2.336	1.337	4.082	0.003
Cardiovascular disease	1.091	2.977	1.840	4.818	<0.001
NLR	0.154	1.167	1.104	1.233	<0.001
LMR	−0.068	0.934	0.812	1.075	0.341
PLR	0.001	1.001	1.000	1.002	0.094
SII, per 100-unit increase	0.054	1.056	1.033	1.079	<0.001
PIV, per 100-unit increase	0.062	1.064	1.038	1.091	<0.001

β, regression coefficient; OR, odds ratio; CI, confidence interval; NLR, neutrophil-to-lymphocyte ratio; LMR, lymphocyte-to-monocyte ratio; PLR, platelet-to-lymphocyte ratio; SII, systemic immune-inflammation index; PIV, pan-immune-inflammation value.

**Table 5:** Multivariate logistic regression model including NLR at admission for catheter-related bloodstream infection in hemodialysis patients.

	Multivariate Analysis				
	β	OR	95% CI		*p*
			Lower	Upper	
Age	−0.022	0.978	0.959	0.998	0.032
Female sex	0.070	1.072	0.624	1.842	0.801
Hypertension	0.893	2.441	1.177	5.066	0.017
Cardiovascular disease	0.965	2.626	1.490	4.629	<0.001
NLR	0.148	1.159	1.099	1.223	<0.001

β, regression coefficient; OR, odds ratio; CI, confidence interval; NLR, neutrophil-to-lymphocyte ratio. Nagelkerke R^2^ = 0.322.

**Table 6:** Multivariate logistic regression model including SII at admission for catheter-related bloodstream infection in hemodialysis patients.

	Multivariate Analysis				
	β	OR	95% CI		*p*
			Lower	Upper	
Age	−0.018	0.982	0.964	1.001	0.059
Female sex	0.048	1.049	0.619	1.777	0.860
Hypertension	0.700	2.014	1.012	4.011	0.046
Cardiovascular disease	0.994	2.702	1.556	4.692	<0.001
SII, per 100-unit increase	0.054	1.055	1.032	1.079	<0.001

β, regression coefficient; OR, odds ratio; CI, confidence interval; SII, systemic immune-inflammation index. Nagelkerke R^2^ = 0.259.

**Table 7:** Multivariate logistic regression model including PIV at admission for catheter-related bloodstream infection in hemodialysis patients.

	Multivariate Analysis				
	β	OR	95% CI		*p*
			Lower	Upper	
Age	−0.018	0.982	0.964	1.001	0.068
Female sex	0.154	1.167	0.685	1.987	0.570
Hypertension	0.791	2.205	1.080	4.502	0.030
Cardiovascular disease	1.004	2.730	1.558	4.782	<0.001
PIV, per 100-unit increase	0.061	1.063	1.036	1.090	<0.001

β, regression coefficient; OR, odds ratio; CI, confidence interval; PIV, pan-immune-inflammation value. Nagelkerke R^2^ = 0.292.

## Data Availability

The data underlying this article are available from the corresponding author upon reasonable request, subject to institutional and ethical restrictions.

## Abbreviations

The following abbreviations are used in this manuscript:

AUC	area under the curve
CBC	complete blood count
CI	confidence interval
CRBSI	catheter-related bloodstream infection
CRP	C-reactive protein
HD	hemodialysis
IQR	interquartile range
LMR	lymphocyte-to-monocyte ratio
MPV	mean platelet volume
NLR	neutrophil-to-lymphocyte ratio
OR	odds ratio
PIV	pan-immune-inflammation value
PLR	platelet-to-lymphocyte ratio
ROC	receiver operating characteristic
SII	systemic immune-inflammation index
SPSS	Statistical Package for the Social Sciences
VIF	variance inflation factor
WBC	white blood cell count
